# A Label-Free Optical Biosensor Based on an Array of Microring Resonators for the Detection of Human Serum Albumin

**DOI:** 10.3390/s24020677

**Published:** 2024-01-21

**Authors:** Xin Chen, Mingyu Li, Zhaoyu Wang, Kaihao Zhao, Jiamei Gu, Qiushun Li, Jian-Jun He

**Affiliations:** 1 Department of Optical Engineering, School of Opto-Electronic Engineering, Changchun University of Science and Technology, Changchun 130022, China; 2021100229@mails.cust.edu.cn (X.C.); wang.zhaoyu@mails.cust.edu.cn (Z.W.); 2021100278@mails.cust.edu.cn (K.Z.); 2 State Key Laboratory of Modern Optical Instrumentation, College of Optical Science and Engineering, Zhejiang University, Hangzhou 310027, China; gu_jiamei@zju.edu.cn (J.G.); jjhe@zju.edu.cn (J.-J.H.); 3 Dezhou Research Institute, Qilu University of Technology (Shandong Academy of Sciences), Dezhou 253084, China

**Keywords:** array of microring resonators, label-free detection, surface functionalization, HSA protein

## Abstract

We introduced a label-free sensing system based on an array of microring resonators (MRRs) which was successfully employed for human serum albumin (HSA) detection. The sensing-ring surface was functionalized to immobilize anti-HSA, facilitating HSA binding. Our refractive index sensing system demonstrates high sensitivity at 168 nm/RIU and a low limit of detection (LOD) of 63.54 ng/mL, closely comparable to current HSA detection methods. These findings confirm the potential of MRRs as biocompatible sensors for HSA detection. This system holds great promise as an innovative platform for the detection of HSA, carrying significant importance in medical diagnostics.

## 1. Introduction

Human serum albumin (HSA) is the most abundant natural carrier protein in plasma (constituting 60% of the overall plasma protein), which is synthesized only in the liver [1]. The serum albumin concentration is about 35–50 g/L, and the urine albumin concentration is usually less than 30 mg/L [2]. HSA is a valuable biomarker of many diseases, including cancer, rheumatoid arthritis, ischemia, post-menopausal obesity, and diseases that need the monitoring of glycemic control [3]. It is foreseen that changes in the HSA occur in tandem with some diseases, presenting a prospect for these alterations to serve as potential biomarkers [4]. Thus, achieving the precise and timely detection of HSA holds significant importance.

Currently, to assess the sensitivity and range of HSA, a variety of analytical methods are employed. These include mass spectrometry [5], fluorescence spectroscopy [6], the enzyme-linked immunosorbent assay (ELISA) approach [7], and the electrochemical method [8]. Mass spectrometry is a powerful tool for the discovery and the analysis of HSA, but it exhibits limitations in terms of accuracy [5,9]. Fluorescence spectroscopy uses various fluorophores to detect HSA, but the limit of detection is high [10,11]. The ELISA approach uses an enzyme-labeled antigen or antibody to quantitatively or specifically identify specific antibodies [12,13]. The practical working range was estimated to be 31.2–2000 ng/mL, achieving a limit of detection below 30 ng/mL [7,14]. The drawback lies in the requirement for an additional label on the analyte. Biomarkers are typically measured using off-chip methods, requiring sampling at specific time points [15,16,17]. However, these repetitive operations could disrupt microtissue responses in the designated microenvironments [18]. As for the electrochemical method, it is a promising approach for detecting HSA due to its speed and high sensitivity. However, a notable disadvantage is the inability to monitor in real time. Additionally, the surface needs to be functional and constructed with a special material [19,20].

In comparison, an MRR sensor based on SOI can be made very small because silicon has a very high refractive index. It gives a very high evanescent field on the surface, so it gives a very high response and very high sensitivity to the sensor [21]. High index contrast allows us to bend the waveguide very tightly with a radius of a few micrometers, so that we can fabricate a sensor that has a long interaction length to give a high response but is still very small. The waveguide can be wrapped around a small area; thus, the sensors can be made into a large array for different functions. Not only can the different types of molecules be detected simultaneously, but also some sensors are used as a reference for monitoring temperature change, monitoring fluidic index change, and distinguishing from a sensing signal [22,23,24]. The most attractive aspect is that MRRs can achieve label-free detection without requiring any additional analyte modification. Table 1 shows a comparison of previous HSA detection methods.

In this paper, our primary emphasis lies in exploring the potential of silicon on insulator (SOI)-based ring resonators for detecting HSA protein. We delved into the silicon surface functionalization methodology to immobilize anti-HSA and achieve the binding of HSA. Notably, we employed an identical set of three sensing-ring arrays in a sensor system utilizing the wavelength interrogation system. In experiments, we effectively introduced modifications to enable the detection of the HSA protein. We evaluated the sensor’s sensitivity (S) at 168 nm/RIU and established its detection limit (DL) at 63.54 ng/mL, closely comparable to ELISA but achieved through a label-free approach. Our high sensitivity, rapid detection, and portability also surpass other methods for HSA detection. Therefore, the MRR holds promise as an innovative platform to meet the demand for detecting HSA.

## 2. Ring Resonators and Sensing Mechanisms

**Figure 1:**
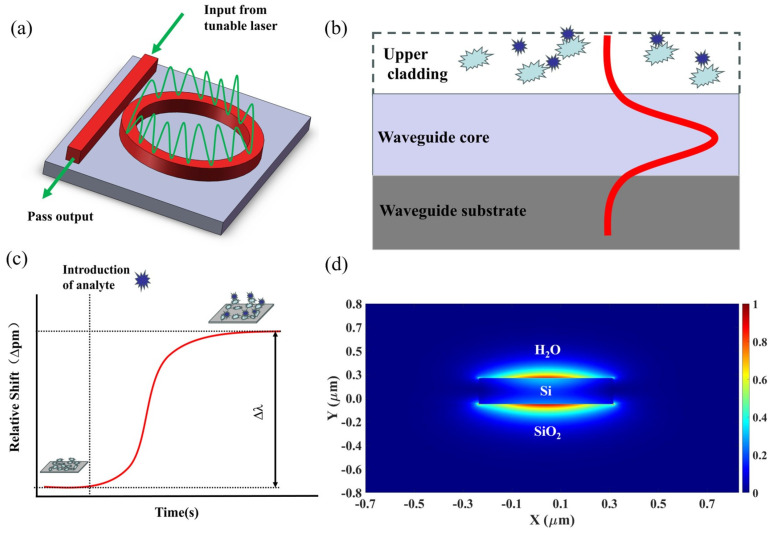
(**a**) Schematic image of the MRR sensors’ optical energy transmission; (**b**) schematic diagram of the principle of evanescent wave sensing; (**c**) relative shift changes observed upon analyte binding on the silicon surface; (**d**) electric field intensity of strip waveguide TM mode.

Silicon photonic microring resonators are integrated microcavity sensors on a chip. Light is coupled into the resonant microcavity via a grating coupler under relevant optical resonance conditions [21]. Figure 1a shows a schematic image of MRR sensors’ optical energy transmission. The resonance condition is determined by the following equation:

(1) $$m\lambda_{r}=2\pi Rn_{eff}$$

where *m* is an integer value, $\lambda_{r}$ is the wavelength of the input light, *R* is the radius of the microring, and $n_{eff}$ is the effective refractive index of the waveguide mode.

In this equation, it becomes evident that variations in the effective refractive index of the optical mode will directly impact the resonance condition’s wavelength ($\lambda_{r}$). This relationship can be succinctly expressed in terms of the relevant variables as follows:

(2) $$\frac{\lambda_{r}}{∆\lambda_{r}}=\frac{n_{eff}}{∆n_{eff}}$$

This relationship emphasizes that even a slight relative change in $n_{eff}$ must correspond to a proportionately small shift in the resonance wavelength [25]. As light propagates through a waveguide, the evanescent wave interacts with the surroundings, as shown in Figure 1b. The change in the evanescent wave affects the waveguide’s effective refractive index, causing a measurable shift in the resonant wavelength. Functionalizing the sensor with capture agents allows the real-time detection and quantification of analytes by monitoring changes in the resonance wavelength due to the altered local refractive index [26,27], as shown in Figure 1c.

In consideration of optical loss, existing measurement instruments, microfluidic channel structures, and process precision, we ultimately opted for strip waveguides. Simulating the transverse magnetic (TM) mode in a water-cladding waveguide using the finite difference eigenmode (FDE) method reveals in Figure 1d that TM-polarized light offers high sensitivity, because a majority of its electric field energy is distributed in the upper cladding, where the analyte is located when the sensors are in operation.

## 3. Chip-Testing Experiment

### 3.1. Parameters of the MRR Chip

**Figure 2:**
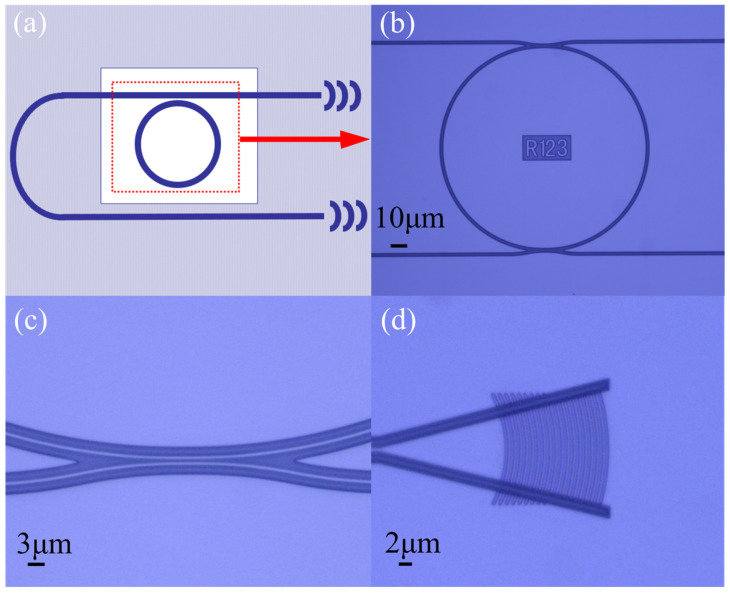
(**a**) Schematic image of the MRR sensor; (**b**) optical microscope image of sensor microrings; (**c**) optical microscope image of the directional coupler; (**d**) optical microscope image of grating coupling.

In the SOI chip, the height and width of our waveguide were set as 220 nm and 500 nm. A schematic image of the MRR sensor is shown in Figure 2a. We utilized 2 μm thick SU8 as the upper cladding. To expose the sensing ring to the analyte sample, we selectively removed the upper cladding layer in the sensing window. Figure 2b–d present optical microscope images of the ring, directional coupler gap, and grating coupler, respectively. The measured radius of the sensing ring is 123 μm, as shown in Figure 2b. In Figure 2c, it is shown that the directional coupler exhibits a gap width of 500 nm. In Figure 2d, one can observe the optical microscope image of the TM grating coupler, featuring an etch depth of 70 nm, a period of 1 μm, and a duty cycle of 50%.

### 3.2. Constructing a Sensor System

**Figure 3:**
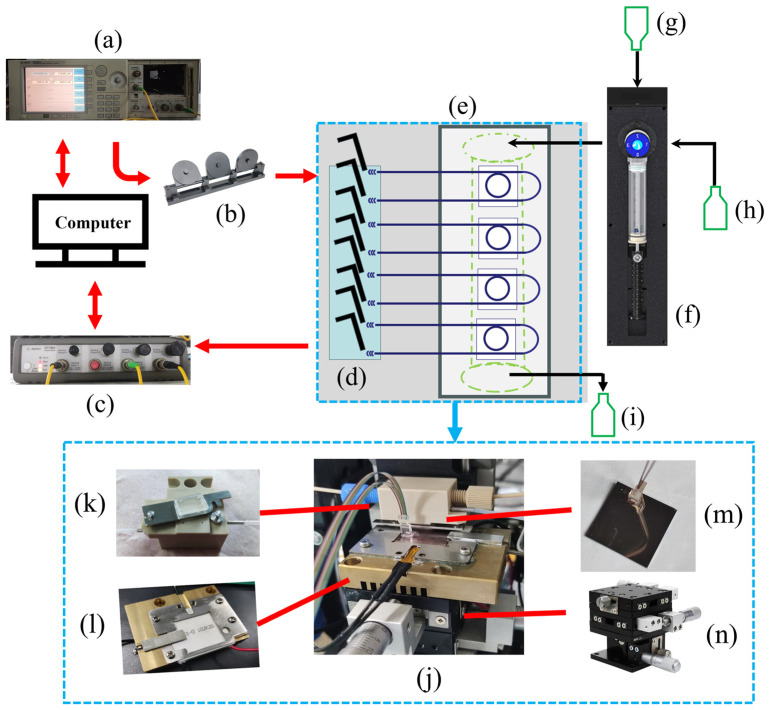
(**a**) Tunable laser, wavelength range of 1500–1600 nm; (**b**) polarization controller; (**c**) photoelectric detector; (**d**) fiber array; (**e**) microfluidic channel system; (**f**) the syringe pump; (**g**–**i**) glass bottle for sample, waste liquid storage, and background liquid; (**j**) photograph of the microfluidic channel system; (**k**) microfluidic channel; (**l**) TEC and heat dissipation brass block; (**m**) chip with fiber array; (**n**) multidimensional translation platform.

The schematic of the sensor system used for performing the wavelength interrogation is shown in Figure 3. All the measurement instruments of the sensing systems were controlled by the computer. A tunable light source (Agilent 81600B, Keysight Technologies, Santa Clara, CA, USA) with a wavelength range of 1500–1600 nm was used as the incident light. The light passed through the polarization controller and entered the waveguide in TM mode via a fiber array. The chip was bonded to the fiber array by using UV-cured adhesive, and the sensing region was placed inside the microfluidic channel structures. Finally, the output light was collected by the power detector (Agilent 7748A, Keysight Technologies, Santa Clara, CA, USA), which had four input ports to meet our requirements. The sensing signals of the wavelength interrogation were processed by the computer.

### 3.3. Results of the Volumetric Refractive Index Sensing Performance

**Figure 4:**
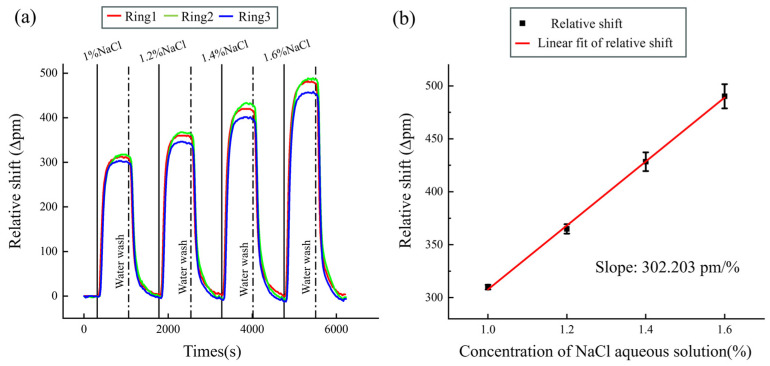
(**a**) Real-time relative shift observed upon introducing varying concentrations of NaCl aqueous solution; (**b**) the function curve of relative shift with the concentration of the NaCl aqueous solution.

The volumetric refractive index sensing performance of the sensor was examined through experiments involving a gradient concentration of NaCl solution. For the wavelength interrogation, a tunable laser and photonic detector were used in the experiment. The signal of the power detector (PD) was then collected in the computer, and the relative shift at a specific wavelength around 1550 nm was analyzed. When we used a microfluidic channel to flow 1%, 1.2%, 1.4%, and 1.6% NaCl aqueous solutions, respectively, through the system, the three sensing rings all exhibited similar relative shifts; the shifts in the spectrum were monitored, and the final result is shown in Figure 4a. We calculated the three rings’ average relative shift and conducted two repeated experiments for accuracy. The collected and averaged results can be fitted with a linear curve, as shown in Figure 4b. Error bars were obtained from three groups of NaCl aqueous solution for each concentration.

The sensitivity of the MRR array system was measured to be S = 302.203 pm/% by extracting the slopes of the curves; we finally measured the array system’s sensitivity to be approximately 168 nm/RIU, corresponding to a refractive index change of about 1.8 × 10^−3^ RIU/% [28]. The refractive index sensor’s limit of detection was calculated as LOD = 3σ/S = 3.98 × 10^−5^ RIU, where the value of standard deviation (σ) obtained from DI water measurements was 2.23 pm.

## 4. Experimental Result and Discussion

### 4.1. Experiment Materials

Anti-human serum albumin antibody (anti-HSA), native human serum albumin (HSA), and human chorionic gonadotropin (HCG) protein were purchased from abcam. Immunoglobulin G (IgG) protein and (3-Aminopropyl) triethoxysilane (APTES) were purchased from Sigma-Aldrich (St. Louis, MO, USA). Phosphate-buffered saline (PBS) was purchased from HyClone (Logan, UT, USA). Bovine serum albumin (BSA), 1-Ethyl-3-(3-dimethylaminopropyl) carbodiimide (EDC), N-Hydroxysuccinimide (NHS), triethylamine (TEA), tetrahydrofuran (THF), polyamidoamine (PAMAM), and all the other reagents were purchased from Macklin Biochemical Co., Ltd. (Shanghai, China).

### 4.2. Surface Functionalization and HSA Detection

**Figure 5:**
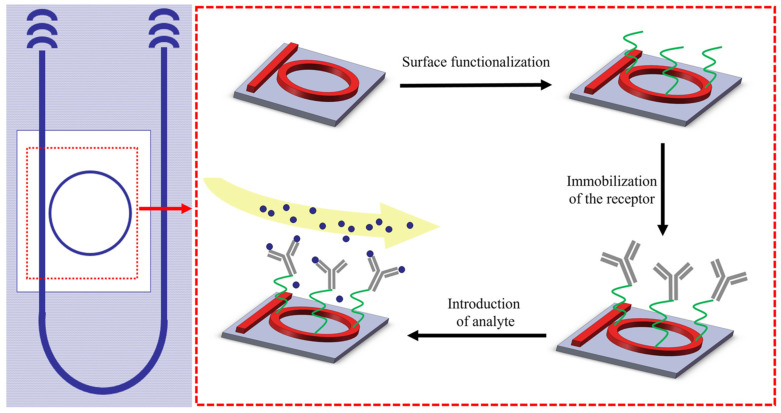
Schematic illustration of silicon chip surface functionalization, the immobilization of the receptor, and the process of analyte detection.

As shown in Figure 5, we functionalized the sensing ring to analyze the special protein. The following is the detailed process of surface functionalization:

(1) Surface functionalization on silicon. The chip was cleaned in isopropyl alcohol by ultrasonication for 15 min followed by rinsing with deionized (DI) water and drying under a stream of N_2_. Silanized chips in a 2% APTES solution were submerged in ethanol for 3 h; then, we proceeded to bake the chips at 120 °C for one hour. Then, we weighed 0.1917 g of succinic anhydride and dissolved it in 250 μL of TEA and 4.75 mL of THF for 4 h, followed by cleaning the sensing region. We prepared a 200 mM EDC and 500 mM NHS cross-linker solution in 5 mL of 100 mM 2-(N-morpholino) ethanesulfonic acid (MES) buffer solution (pH 5.0). Then, we immersed the chips in the solution for 30 min, followed by cleaning once the time was over. We dropped PAMAM solution onto the sensing region in a darkroom at room temperature for 12 h. Afterward, we immersed the chips in methanol and subjected them to ultrasonic treatment for 15 min. We exposed the chips to a 10% glutaraldehyde solution for 30 min.
(2) We achieved the immobilization of the receptor by placing 1 mg/mL anti-HSA onto the sensing region. Then, we transferred the chips to a 4 °C location and let them rest for 12 h. Afterward, we washed off the unbound antibody by rinsing with 10 mM phosphate-buffered saline (PBS). Then, we incubated them with 1% bovine serum albumin (BSA) in PBS at room temperature for 1 h to prevent non-specific protein binding. Finally, we cleaned the sample with 10 mM PBS wash and dried it using nitrogen.

After functionalizing the surface, we introduced the analyte for detection. We prepared 10 μL of 1 mg/mL standard HSA protein solution and sufficient running buffer (10 mM PBS, 0.5% BSA). We remembered to gently vortex each solution prior to use. The protein was diluted from standard HSA protein solution to the required concentrations for our experiment: 78 ng/mL, 156 ng/mL, 312 ng/mL, 625 ng/mL, 1.25 μg/mL, 5 μg/mL, and 10 μg/mL. After diluting the protein, we incubated the samples at room temperature until they reached ambient temperature before commencing the experimental procedure. After completing all the steps, we loaded the pre-functionalized chips into the microfluidic channel system. An initial running buffer rinse (PBS, 0.5% BSA) was performed to establish a baseline for 30 min while commencing optical monitoring of the resonance wavelength shift. Subsequently, we introduced the first HSA sample and allowed it to flow for 10 min, followed by a running buffer rinse step, which typically ranges from 5 to 10 min. The remaining reagents were then introduced in sequential steps. The liquid flow rate for both the sample and running buffer was 30 μL/min. Remarkably, the upper cladding layer confined the surface functionalization to the openings surrounding the sensing region, ensuring that the signal of the relative shift originated solely from the sensing ring. Once the experiment was completed, we thoroughly rinsed the fluid lines with deionized water.

### 4.3. Result and Discussion

**Figure 6:**
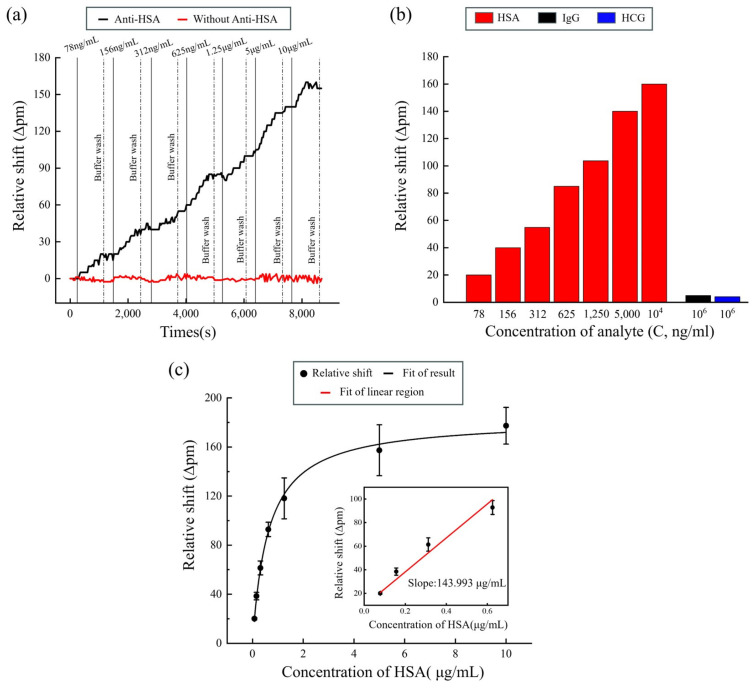
(**a**) The dynamic variation in the relative shift of the anti-HSA sensors and sensors without anti-HSA; (**b**) the relative shift of different solutions for the HSA, IgG, and HCG protein; (**c**) fit of the experimental results of the anti-HSA sensors.

In the experiment, we first carried out silicon surface functionalization and then immobilized the anti-HSA receptor. Subsequently, we assessed the response to the HSA sample concentration. For quantitative detection, once the baseline stabilized, indicated by a relative shift of 0 over a certain period, various concentrations of HSA aqueous solution were introduced into the microfluidic channel. The samples were injected for 10 min at a flow rate of 30 μL/min and subsequently rinsed with a running buffer (PBS, 0.5% BSA). Additionally, by introducing the required gradient proteins onto the surface of the sensors without anti-HSA during the experiment, we aimed to verify whether the spectral shift originated from changes in concentration. Figure 6a displays the dynamic variation in the relative shift over time in the anti-HSA sensors and sensors without anti-HSA. In anti-HSA sensors, the relative shift gradually increases with the concentrations of HSA, exhibiting concentration-dependent binding of HSA. In sensors without anti-HSA, the results show that the spectrum does not exhibit significant drift, indicating that the observed relative shift solely arises from the binding of HSA proteins. To verify specific binding, we conducted a control experiment measuring human chorionic gonadotropin (HCG) and immunoglobulin G (IgG) proteins, which exhibited a much lower response (less than 5 pm at 10^6^ ng/mL of HCG and 1.25 × 10^6^ ng/mL of IgG) compared to the specific protein binding, as illustrated in Figure 6b. Consequently, our proposed sensor exhibits excellent selectivity for the detection of HSA.

A concentration of 156 ng/mL was selected for a more detailed study, with standard deviations (*n* = 3) obtained for all the antigen concentrations measured. The standard deviation σ is 3.05 pm; thus, the noise level is N = 3σ = 9.15 pm. In Figure 6c, we fit the relative shift with concentrations across the entire range of the analyte solution, resulting in an R-squared value of 0.997, with the error bars showing the difference in the three rings. Additionally, we found a linear relationship of the sensor response (∆λ = 9.4717 + 143.993 × C, R^2^ = 0.9468) within the range of 78 ng/mL to 625 ng/mL of analyte. The slope of the fitting line is S = 143.993 pm/(μg·mL^−1^). Therefore, the LOD was calculated to be 63.54 ng/mL, which is determined as the concentration of the sample with the response equal to the noise level [22]. In the experiments, the MRR array system detected HSA in the range of 63.54 ng/mL to 625 ng/mL. After introducing a protein concentration of 1.25 μg/mL, a gradual decrease in the slope occurs as the binding between anti-HSA and HSA approaches saturation. At 10 μg/mL, the anti-HSA immobilized on the silicon surface almost entirely binds with the HSA protein, and the fitted curve gradually levels off. By choosing an improved surface functionalization to immobilize more antibodies, the detection range can be enlarged. This introduces an approach for advancing HSA detection methodologies.

## 5. Conclusions

In summary, we report a refractive index sensing system based on an SOI microring resonator array to detect HSA successfully by functionalizing the silicon surface. A high sensitivity of 168 nm/RIU, along with a low limit of detection of 63.54 ng/mL and detection range from 63.54 ng/mL to 625 ng/mL, was achieved. We accomplished the HSA detection without an additional label, and our results closely resemble conventional HSA detection, demonstrating significant potential in the medical clinical detection of HSA. In the future, we will explore advanced surface modification methods to immobilize more anti-HSA on a silicon-based surface, thereby expanding the sensor’s detection range. Additionally, we aim to optimize the experimental system to enhance its stability and portability. This involves replacing wavelength interrogation with intensity interrogation, utilizing a broadband source. The LED and Ge detector can be integrated on a silicon waveguide sensor chip in order to further miniaturize the system and increase the portability. The advantages of our sensor system, combined with its low detection limits and inherent multiplexing capability, position SOI microring resonators as a promising platform for label-free biomolecular analysis, particularly for the detection of human proteins related to human health.

## Figures and Tables

**Table 1:** Comparison of previous HSA detection methods.

Detection Methods	Detection Range	LOD	Label	Reference
Mass spectrometry	10.5–625 mg/L	4.84 mg/L	Yes	[5]
Fluorescence spectroscopy	10–320 mg/L	5.5 mg/L	Yes	[6]
ELISA approach	31.2–2000 ng/mL	7.8 ng/mL	Yes	[7]
Electrochemical method	2.5–500 µg/mL	1.55 µg/mL	No	[8]
MRR	63.54–625 ng/mL	63.54 ng/mL	No	This work

## Data Availability

Data are contained within the article.
